# Urinary *PCA3* detection in prostate cancer by magnetic nanoparticles coupled with colorimetric enzyme-linked oligonucleotide assay 

**DOI:** 10.17179/excli2020-1036

**Published:** 2020-04-15

**Authors:** Vichanan Yamkamon, Khin Phyu Pyar Htoo, Sakda Yainoy, Thummaruk Suksrichavalit, Tienrat Tangchaikeeree, Warawan Eiamphungporn

**Affiliations:** 1 Department of Clinical Microscopy, Faculty of Medical Technology, Mahidol University, Bangkok, Thailand; 2 Department of Clinical Microbiology and Applied Technology, Faculty of Medical Technology, Mahidol University, Bangkok, Thailand; 3 Department of Medical Laboratory Technology, University of Medical Technology, Mandalay, Myanmar; 4 Center of Data Mining and Biomedical Informatics, Faculty of Medical Technology, Mahidol University, Bangkok, Thailand; 5 Center for Research and Innovation, Faculty of Medical Technology, Mahidol University, Bangkok, Thailand

**Keywords:** PCA3, prostate cancer, urine, magnetic nanoparticle, colorimetric assay

## Abstract

*PCA3* is one of the most prostate cancer-specific genes described to date. Of note, *PCA3* expression is detectable at high level in the urine of prostate cancer (PCa) patients. Accordingly, *PCA3* is an ideal biomarker for PCa diagnosis. Several techniques for the measurement of this biomarker in urine have been developed but there are still some drawbacks. In this study, magnetic nanoparticle-based PCR coupled with streptavidin-horseradish peroxidase and a substrate for colorimetric detection was established as a potential assay for urinary *PCA3* detection. The method provided a high specificity for *PCA3* gene in LNCaP prostate cancer cell line. Additionally, this technique could detect *PCA3 *at femtogram level which was approximately 1,000-fold more sensitive than the conventional RT-PCR followed by agarose gel electrophoresis. The effectiveness of the method was assessed by *PCA3* detection in clinical specimens. The relative *PCA3* expression of PCa patients determined by this assay was significantly greater than that of benign prostatic hyperplasia (BPH) patients and healthy controls. The results of our test were comparable with the results of qRT-PCR. The proposed method is promising to distinguish between cancerous and non-cancerous groups. Altogether, this simple assay is practicable and useful for prostate cancer diagnosis.

## Introduction

Prostate cancer (PCa) is the most common type of cancer diagnosed in men and a leading cause of cancer-related deaths (Ma and Huang, 2017[19]). The incidence of prostate cancer has been reported in speedily growing trend worldwide (Siegel et al., 2018[30]; Taitt, 2018[33]). In clinical practice, prostate specific antigen (PSA) blood test combined with digital rectal examination (DRE) is currently used for PCa screening and monitoring (Smith et al., 2004[31]). Based on suspicious DRE and/or increased PSA level in serum, biopsy which is more invasive is manipulated (Catalona et al., 1994[7]). A serum PSA level of below 4 ng/mL is considered normal and it increases significantly in men with prostate cancer. Nevertheless, this biomarker is specific to tissue of prostate origin but not only in PCa, PSA level is often elevated in benign prostatic hyperplasia (BPH) and prostatitis (Roobol et al., 2007[27]). Moreover, previous studies indicated 15 % of PCa patients have PSA level below 4.0 ng/mL which leads to false negative results (Thompson et al., 2004[36]). As a result of limited specificity of PSA, role of PSA for PCa diagnosis still remains controversial (Cucchiara et al., 2018[9]; Catalona, 2018[6]). Currently, the United States Preventive Services Task Force (USPSTF) has new guidelines that PSA test may be less beneficial for some men and it should not be screened routinely (US Preventive Services Task Force et al., 2018[37]). Therefore, the new finding of more specific PCa markers for screening and diagnosis is essential.

Prostate cancer antigen 3 (*PCA3*), also known as differential display code 3 or *DD3*, is a non-coding RNA and it is overexpressed in 95 % of primary PCa tissue specimen (Bussemakers et al., 1999[5]). However, it was undetectable in bladder, breast, cervix, endometrium, kidney, ovary or testis tumors (Loeb and Partin, 2011[18]). Notably, the expression of *PCA3* in BPH and normal tissues was very low (Wang et al., 2014[38]). Interestingly, *PCA3 *can be detected in post DRE urine samples, thus it was proposed as a promising biomarker for PCa. The US Food and Drug Administration (FDA) recently approved Progensa *PCA3 *assay as the first urine-based molecular test for clinical diagnosis recommended for men with elevated serum PSA and a previous negative biopsy (Cui et al., 2016[10]). *PCA3* assay is commercially available, but it is expensive and time-consuming as well as it needs a complicated instrument. To overcome these limitations, a simple, sensitive and cost-effective assay for *PCA3* detection in urine samples is absolutely crucial.

In the last two decades, nanotechnology and nanoscience have been greatly employed in various fields, such as molecular diagnostics, biomedicine, bioseparation and drug delivery (Akbarzadeh et al., 2012[1]; Mody et al., 2014[21]). Particularly, nanoparticles (NPs) have been used as signal amplification tools due to their small size (1-100 nm), corresponding high surface-to-volume ratio and unusual target binding properties (Perez et al., 2011[24]). Among the broad spectrum of nanoparticles, magnetic nanoparticles (MNPs) are a major class of nano-scale material currently under extensive development for a wide range of applications (Shabestari Khiabani et al., 2017[29]). MNPs have been exploited because of their exclusive physical properties, magnetic susceptibility, biocompatibility, stability, easy functionalization and many more relevant features (Amiri et al., 2019[2]). MNPs functionalized with targeting moieties enable the efficient collection and separation of target molecules in a simple and rapid process without any filtration or centrifugation (Kim and Searson, 2017[16]). Recently, MNP-based polymerase chain reaction (PCR) coupled with colorimetric assay has been developed for gene detection of several diseases (Jangpatarapongsa et al., 2011[14]; Tangchaikeeree et al., 2017[34]). This strategy combines the versatility of colorimetric assay based on enzyme-substrate system and the exponential amplification capacity of PCR using MNPs functionalized with oligonucleotide primer. The PCR method is a highly sensitive and specific technique that can amplify a huge number of DNA copies from a single target DNA, allowing an effective detection from a small amount of sample. Magnetic field has been applied for isolation of nucleic acid after amplification to increase the sensitivity of the test. Colorimetric assay is a simple and sensitive method that can be visually detected or requires only a spectrophotometer. Taken together, this methodology is facile, highly specific and provides better sensitivity when compared with the conventional RT-PCR followed by agarose gel electrophoresis.

**Figure 1:**
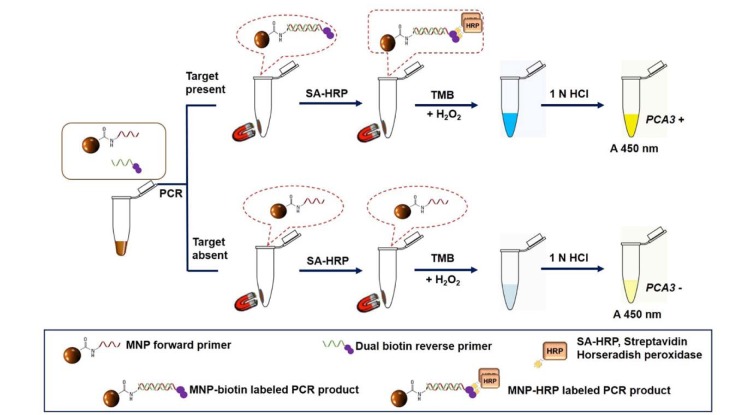
A schematic illustration of the MNP-based PCR coupled with colorimetric enzyme-linked oligonucleotide assay for *PCA3* detection in urine sediments

In this study, the MNP-based PCR coupled with colorimetric enzyme-linked oligonucleotide assay was developed to detect urinary *PCA3* for PCa determination. This technique based on RT-PCR using MNP-labeled forward primer and dual biotin-labeled reverse primer combined with horseradish peroxidase-streptavidin detection system. The schematic diagram of the assay is illustrated in Figure 1(Fig. 1). The proposed method showed high level of analytical sensitivity as well as specificity and it could potentially be used for the detection of *PCA3*. Moreover, this methodology could differentiate PCa patients from both healthy controls and BPH patients based on *PCA3 *level in urine. In conclusion, our assay holds a promising prospect for PCa diagnosis.

## Materials and Methods

### Materials and reagents

MNPs were synthesized by co-precipitation of FeCl_3_ and FeCl_2_, then they were carboxylate-functionalized by emulsion polymerization according to previously described procedures (Montagne et al., 2002[22]; Braconnot et al., 2013[4]). For MNP immobilization, all specific primers including amino (NH_2_)-labeled forward and dual biotin-labeled reverse primers were ordered from Integrated DNA Technologies, Inc. (Skokie, IL, USA). All chemicals required for the immobilization were from Sigma Aldrich (St. Louis, MO, USA). SsoAdvanced Universal SYBR Green Supermix was purchased from Bio-Rad (Hercules, CA, USA). RevertAid First Strand cDNA synthesis kit and Phusion high-fidelity DNA polymerase were obtained from Thermo Fisher Scientific (Waltham, MA, USA). Media and reagents used for cell culture were purchased from Hyclone (Logan, UT, USA) and Gibco (Carlsbad, CA, USA).

### Cell lines 

LNCaP clone FGC (ATCC CRL-1740) prostate cancer cell line was used as a positive control for *PCA3*. RWPE-1 (ATCC CRL-11609) prostate epithelial cell line, MDA-MB-436 (ATCC HTB-130) breast cancer cell line and K562 (ATCC CCL-243) chronic myelogenous leukemia cell line were used as negative controls for *PCA3*. All cell lines were purchased from the American Type Culture Collection (ATCC, Rockville, MD, USA). They were maintained in defined media and cultured under the conditions as previously described (Htoo et al., 2019[13]; Promkan et al., 2013[25]; Suangtamai and Tanyong, 2016[32]).

### Urine sample collection and preparation

Spot urine samples were collected from healthy male subjects (n = 5) and first voided post-DRE urine samples were received from BPH patients (n = 5) as well as from PCa patients (n = 5). All patients were diagnosed based on histopathological analysis after prostate biopsy. Samples were provided by the Division of Urology, Department of Surgery, Faculty of Medicine, Ramathibodi Hospital, Mahidol University. The present study was approved by the Committee on Human Rights Related to Research Involving Human Subjects, Faculty of Medicine, Ramathibodi Hospital, Mahidol University (MURA2016/34). Written informed consent was obtained from each individual in the study. After collection, urine samples were centrifuged at 4 °C, 3,000 rpm for 10 min. The supernatants were discarded and cell pellets were washed twice by PBS, pH 7.0. Subsequently, TRIzol reagent (Invitrogen, CA, USA) was added to the sediments which were then stored at -20 °C until RNA isolation.

### RNA extraction and cDNA synthesis

Total RNA was isolated from cell lines or from cell sediments of urine with TRIzol reagent. 500 ng of total RNA was converted to cDNA using RevertAid First Strand cDNA synthesis kit following the manufacturer's instructions and kept at -20 °C until use.

### Immobilization of forward primer on MNPs

Forward primer was immobilized on MNPs using carbodiimide crosslinking method as previously described (Nakajima and Ikada, 1995[23]). Briefly, 1 mg of carboxylated MNPs was washed twice with 100 μL of 25 mM 2-(N-morpholino) ethanesulfonic acid (MES) buffer, pH 6.0. The washed MNPs were resuspended in the 50 μL of MES buffer and thoroughly mixed with 5 nmol of NH_2_-modified *PCA3* forward primer. The mixture was incubated at room temperature with gentle shaking for 30 min. The surface of MNPs was subsequently activated by adding a freshly prepared *N*-(3-Dimethylaminopropyl)-*N′*-ethylcarbodiimide (EDC) solution (10 mg/mL) with the ratio of 1:1 (v/v). The activated MNPs were then incubated with shaking at 900 rpm, room temperature for overnight. Using external magnetic field, MNP-immobilized *PCA3* forward primer was separated and supernatant was simultaneously collected. The residual primer concentration in the supernatant was measured at 260 nm using a NanoDrop2000c UV-Vis spectrophotometer (Thermo Fisher Scientific, Waltham, MA, USA). Afterwards, the MNP-labeled forward primer was washed twice and completely resuspended in 50 μL of TE buffer (10 mM Tris-HCl, 1 mM EDTA, pH 8.0). The MNP-labeled primer was stored at 4 °C until use.

### Conventional RT-PCR

*PCA3* amplification was performed using conventional primers. The sequences of primers are shown in Table 1(Tab. 1). PCR was carried out in a 50 µl reaction mixture containing 0.05 µg of cDNA template, 0.5 µM of each primer, 1x HF reaction buffer, 0.2 mM dNTPs and 0.4 U Phusion high-fidelity DNA polymerase. The cycling procedures were performed as followed: initial denaturation at 98 °C for 30 sec followed by 30 cycles of denaturation at 98 °C for 10 s, annealing at 60 °C for 30 s, extension at 72 °C for 30 s and a final extension step at 72 °C for 5 min. Of note, a housekeeping gene, *GADPH* gene was used as a control to evaluate the cDNA quality. To amplify *GADPH*, the cycling procedures were set for 5 min at 95 °C, followed by 30 cycles of 30 s at 95 °C, 30 s at 50 °C, and 30 s at 72 °C, and finally extension 5 min at 72 °C. After amplification, 10 µL PCR products were analyzed by gel electrophoresis. The PCR amplicon sizes of *PCA3* and *GADPH* were 167 bp and 106 bp, respectively. 

### Optimization of primer concentration for MNP-based PCR

Prior to perform the MNP-based PCR, the concentrations of MNP-labeled forward primer were optimized. In brief, PCR was achieved in a 50 µL reaction mixture containing 0.05 µg of cDNA template, 0.5 µM reverse primer, 1× HF reaction buffer, 0.2 mM dNTPs and 0.4 U Phusion high-fidelity DNA polymerase. Each concentration of MNP labeled-forward primer (2, 4 and 6 µg) was individually added to the PCR reaction. The MNP-based PCR amplification was performed with identical conditions of the conventional RT-PCR, as above mentioned.

### MNP-based PCR coupled with colorimetric assay for PCA3 detection

The optimized procedure of MNP-based PCR for *PCA3* detection was performed. The 6 μg MNP-labeled forward primer was applied in the PCR reaction. The PCR was performed with identical conditions of the conventional RT-PCR as previously mentioned. Subsequently, PCR products were detected by colorimetric method. Briefly, the MNP-bound PCR products were washed with deionized water using magnetic separation. After washing, the MNP-bound PCR products were mixed with 50 μL of horseradish peroxidase-conjugated streptavidin (SA-HRP) in 0.1 % BSA and incubated for 30 min in a dark chamber at room temperature. The SA-HRP conjugated PCR products were washed with 100 μL of 0.1 % BSA and then with deionized water. The conjugated PCR products were incubated with 50 μL of SureBlue^TM^ TMB (3, 3', 5, 5'-tetramethylbenzidine) microwell peroxidase substrate (KPL, Gaithersburg, MO, USA) for 10 min in the dark at room temperature. The reaction was stopped by 50 μL of 1 N HCl. The optical density (OD) at 450 nm was measured using microplate reader (BioTek Inc., Winooski, USA). The results were calculated to the relative OD_450_ which means the OD_450_ ratio of the sample to the non-template control (NTC).

### Sensitivity and specificity tests

The analytical sensitivity of the assay was investigated. Serial ten-fold dilutions of cDNA from LNCaP cells ranging from 1 µg to 1 fg were prepared. Each dilution was employed as a template for MNP-based PCR. To examine the analytical specificity, the cDNA samples from LNCaP, RWPE-1, MDA-MB-436 and K562 cell lines were utilized as templates for MNP-based PCR. The PCR product of NTC was used a negative control. The MNP-based PCR products were determined using an enzyme-substrate system in the same manner as described above.

### qRT-PCR

qRT-PCR was achieved as a standard method to determine the expression of target genes. qRT-PCR primers are shown in Table 1(Tab. 1). Each PCR reaction was performed in a 20 μL reaction mixture comprising 1× SsoAdvanced Universal SYBR Green Supermix, 0.5 μM of each primer and 0.025 µg of cDNA template. The qRT-PCR program for *PCA3* and *GAPDH* was set for 3 min at 95 °C, followed by 40 cycles of 20 s at 95 °C and 30 s at 60 °C. The baseline threshold was adjusted and the Ct was determined. Melt curve analysis was analyzed by CFX™ manager software 3.1 (Bio-Rad, Hercules, CA, USA). Gel electrophoresis was conducted to confirm the presence of PCR products. The relative expression level of *PCA3* was calculated by a 2^-ΔΔCt^ relative quantification method according to the manufacturer's instruction.

### Statistical analysis

Data were analyzed by SPSS PASW Statistics 25 (SPSS Inc., Chicago, USA) and GraphPad Prism (version 7.03, GraphPad Software). They were represented as mean ± standard deviation (SD). The differences between groups in each experiment (samples from cell lines and subjects) were compared using one-way ANOVA. Post hoc analysis was applied by using Dunnett's t multiple comparison for testing of sensitivity and specificity. Linear regression method was used to examine the relationship between the different concentrations of the standards and relative absorbance values. Statistical significance was defined as a *P*-value < 0.05.

## Results

### Primer immobilized efficiency

To obtain MNP-labeled forward primer, 5 nmol NH_2_-modified *PCA3* forward primer was immobilized onto the surface of 1 mg carboxylated MNPs using EDC as a coupling agent. The residual *PCA3* forward primer concentration in the supernatant after immobilization was measured to evaluate the binding efficiency. The result demonstrated that the average concentration of the residual *PCA3* forward primer was 2.4 ± 0.02 nmol/ mg corresponding to the immobilized NH_2_-modified *PCA3* forward primer at 2.6 ± 0.02 nmol/mg. The binding efficiency was relatively calculated to 51.9 %.

### Optimization of primer concentration for MNP-based PCR

**Figure 2:**
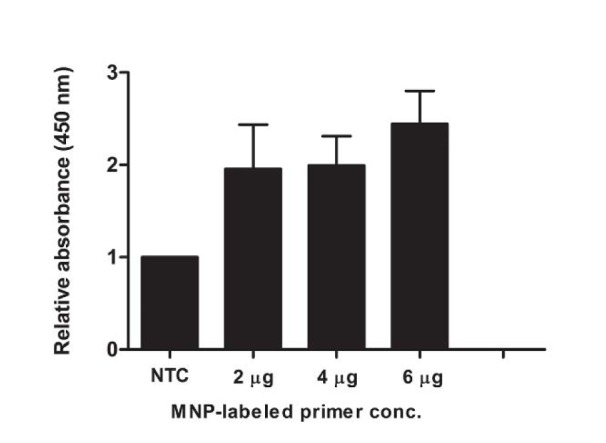
Optimization of primer concentration for MNP-based PCR. Each concentration (2, 4 and 6 µg) of MNP-labeled forward primer was added in the PCR reaction. After MNP-based PCR coupled with colorimetric assay, the reactions were measured the OD_450_. The relative absorbance (450 nm) of each PCR reaction was calculated comparing to the negative control (NTC, no-target control of PCR reaction). Each bar indicates the relative absorbance (mean ± SD).

PCR for *PCA3* amplification was conducted using 10 µM dual biotinylated reverse primer and different concentrations of MNP conjugated-forward primer. As shown in Figure 2(Fig. 2), the sample using 6 μg of MNP conjugated-forward primer exhibited the highest OD_450 _ratio. At this concentration, the relative OD_450 _of positive sample was approximately 3-fold greater than that of negative control.

### Sensitivity test and detection limit

**Figure 3:**
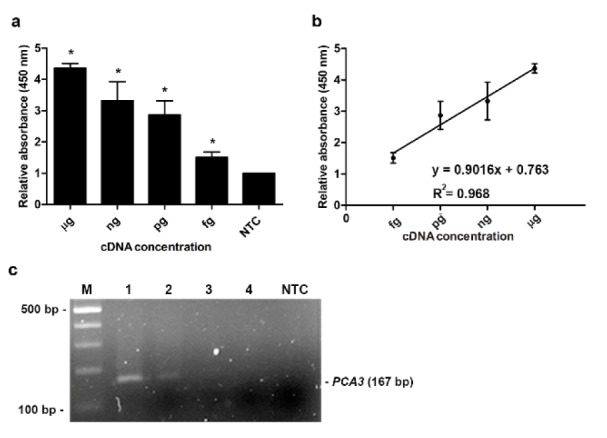
Sensitivity analysis (a) Relative absorbance (450 nm) of the MNP-based PCR coupled with colorimetric assay obtained from the different cDNA template concentrations ranging from 1 µg to 1 fg. Bars represent the relative absorbance (mean ± SD) calculated from the OD_450_ of each sample divided by the OD_450_ of control (NTC; no-target control). The* t*-test analysis was performed and *P-value < 0.05 compared with NTC was indicated. (b) Calibration curve of the relative absorbance at various concentrations of cDNA template. (c) Agarose gel electrophoresis of PCR products obtained from the different cDNA template concentrations. Lane M, 100 bp ladder; Lane 1, 1 µg; Lane 2, 1 ng; Lane 3, 1pg; Lane 4, 1 fg; NTC, no-target control.

The sensitivity of the assay was investigated. Different concentrations of cDNA from LNCaP (1 µg to 1 fg) were employed for MNP-based PCR followed by enzyme-substrate detection system. The same concentrations of cDNA template were also used for conventional RT-PCR. As shown in Figure 3c(Fig. 3), at lower concentration of cDNA (1 pg to 1 fg), no PCR product was visibly observed on gel electrophoresis. Meanwhile, our developed method could detect the PCR products when applying 1 fg of cDNA template (Figure 3a(Fig. 3)). It should be noted that the relative OD_450_ linearly increased corresponding to the increase of cDNA concentration (R^2 ^= 0.968) (Figure 3b(Fig. 3)). As a result, the detection limit of the assay was approximately 1 fg. These results suggested that our assay was more sensitive than the conventional RT-PCR. Interestingly, the relative OD_450_ of positive results (1 µg to 1 fg) was statistically greater than that of NTC (*P*-value < 0.05) (Figure 3a(Fig. 3)). In addition, the OD_450_ ratio of positive results was significantly higher than the cutoff OD_450 _ratio calculated from the OD_450_ ratio of NTC + 3 SD of NTC.

### Specificity test

**Figure 4:**
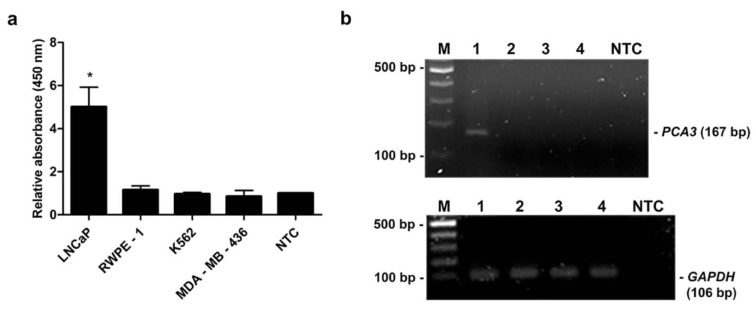
Specificity analysis. (a) Relative absorbance (450 nm) of the MNP-based PCR coupled with colorimetric assay using cDNA template obtained from various cell lines; LNCaP, RWPE-1, K562 and MDA-MB-436. Bars represent the relative absorbance (mean ± SD) calculated from the OD_450_ of each sample divided by the OD_450_ of NTC. The *t*-test analysis was performed and *P-value < 0.05 compared with NTC was indicated. (b) PCR products of *PCA3 *(upper gel) and *GAPDH* (lower gel) analyzed by agarose gel electrophoresis. Amplicon sizes of *PCA3* and *GAPDH* were 167 bp and 106 bp, respectively. Lane M, 100 bp ladder; Lane 1-4, PCR products from LNCaP, RWPE-1, K562 and MDA-MB-436, respectively; Lane NTC, no-target control.

The specificity of primers for *PCA3 *was tested using conventional RT-PCR. The cDNA from various cell lines, i.e., RWPE-1, LNCaP, K562 and MDA-MB-436 was individually applied as a template. Distilled water was used as a negative control (NTC). Of note, the quality of each cDNA was verified by amplification of *GAPDH *gene. PCR products were analyzed by gel electrophoresis. As shown in Figure 4b(Fig. 4), 106 bp PCR products of *GAPDH *were detected from the cDNA of all tested cell lines suggesting the good quality of the cDNA templates. Additionally, 167 bp PCR amplicons of *PCA3 *were detected only from LNCaP cell line, whereas the amplified product was not observed from RWPE-1, K562 and MDA-MB-436 cell lines (Figure 4b(Fig. 4)). Simultaneously, the MNP-based PCR was employed using MNP-labeled forward and dual biotin-labeled reverse primers. PCR products were determined by enzyme-substrate system. As expected, the relative OD_450_ of PCR products using cDNA from LNCaP cell line was significantly greater than that of PCR products from RWPE-1, K562, MDA-MB-436 cell lines and NTC *(P*-value < 0.05) (Figure 4a(Fig. 4)). Notably, the OD_450_ ratio of a positive sample was higher than the OD_450_ ratio from the cutoff OD_450_ ratio of NTC + 3 SD of NTC. These results indicated that the designed primers were very specific to *PCA3* which only expressed in LNCaP prostate cancer cell line.

### Detection of PCA3 in urine using MNP-based PCR

**Figure 5:**
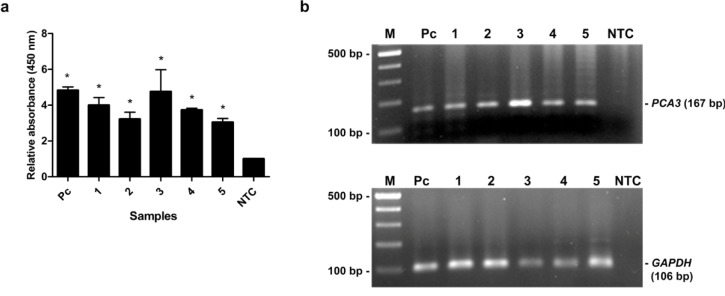
The MNP-based PCR coupled with colorimetric assay of *PCA3* detection from urine of PCa patients. (a) Relative absorbance (450 nm) of each PCa sample (no. 1-5). Bars represent the relative absorbance (mean ± SD) calculated from the OD_450_ of each sample divided by the OD_450_ of NTC. The* t*-test analysis was performed and *P-value < 0.05 compared with NTC was indicated. (b) PCR products of *PCA3 *(upper gel) and *GAPDH* (lower gel) analyzed by agarose gel electrophoresis. Amplicon sizes of *PCA3* and *GAPDH* were 167 bp and 106 bp, respectively. Lane M, 100 bp ladder; Lane 1-5, PCR products from urine of PCa patients; Lane Pc, PCR products from LNCaP cells as a positive control; Lane NTC, no-target control.

**Figure 6:**
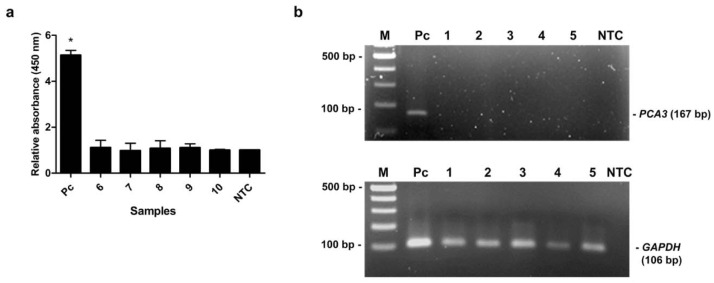
The MNP-based PCR coupled with colorimetric assay of *PCA3* detection from urine of BPH patients. (a) Relative absorbance (450 nm) of each BPH sample (no. 6-10). Bars represent the relative absorbance (mean ± SD) calculated from the OD_450_ of each sample divided by the OD_450 _of NTC. The *t*-test analysis was performed and *P-value < 0.05 compared with NTC was indicated. (b) PCR products of *PCA3 *(upper gel) and *GAPDH* (lower gel) analyzed by agarose gel electrophoresis. Amplicon sizes of *PCA3* and *GAPDH* were 167 bp and 106 bp, respectively. Lane M, 100 bp ladder; Lane 1-5, PCR products from urine of BPH patients; Lane Pc, PCR products from LNCaP cells (positive control); Lane NTC, no-target control.

**Figure 7:**
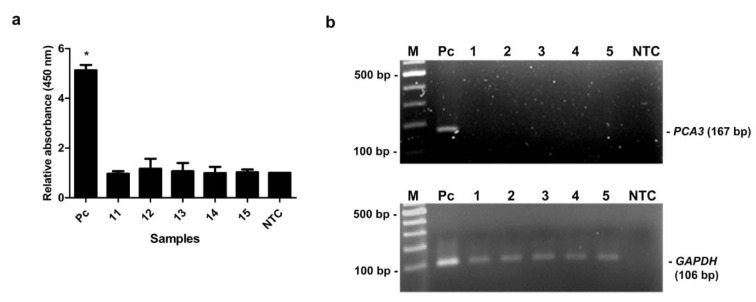
The MNP-based PCR coupled with colorimetric assay of *PCA3* detection from urine of healthy controls. (a) Relative absorbance (450 nm) of each healthy control sample (no. 11-15). Bars represent the relative absorbance (mean ± SD) calculated from the OD_450_ of each sample divided by the OD_450_ of NTC. The *t*-test analysis was performed and *P-value < 0.05 compared with NTC was indicated. (b) PCR products of *PCA3 *(upper gel) and *GAPDH* (lower gel) analyzed by agarose gel electrophoresis. Amplicon sizes of *PCA3* and *GAPDH* were 167 bp and 106 bp, respectively. Lane M, 100 bp ladder; Lane 1-5, PCR products from urine of healthy controls; Lane Pc, PCR products from LNCaP cells (positive control); Lane NTC, no-target control.

The capability of the assay was evaluated by detection of *PCA3* in clinical specimens. The 15 urine samples (5 biopsy-proven PCa patients, 5 BPH patients and 5 healthy subjects) were tested by our method and the results were compared with qRT-PCR results. As shown in Table 2(Tab. 2), the relative *PCA3* expression of PCa patients determined by qRT-PCR was remarkably higher than that of BPH patients and healthy subjects. PCR products of *PCA3* and *GAPDH* were amplified from cDNA of individual by conventional RT-PCR to verify the *PCA3* expression and DNA quality. PCR products of *GAPDH* were amplified from all samples, while *PCA3* products were detected only in PCa patients (Figure 5b(Fig. 5)). No band was visible in other groups (Figure 6b(Fig. 6), 7b(Fig. 7)). Using MNP-based PCR, the results demonstrated that the relative OD_450 _of all PCa samples was statistically higher than that of samples from BPH and healthy groups (P-value < 0.05) (Figure 5a(Fig. 5), 6a(Fig. 6), 7a(Fig. 7)). Furthermore, the relative OD_450_ of all PCa samples was greater than the cutoff OD_450_ ratio of NTC + 3 SD of NTC.

## Discussion

*PCA3* is a long, non-coding prostate-specific RNA, which is highly expressed in prostate neoplasms, but not in benign prostate diseases, such as BPH, prostatitis, and prostatic intraepithelial neoplasia (PIN) (Deras et al., 2008[11]). Intriguingly, this biomarker presents in urine. Use of the Progensa *PCA3 *commercial test for post-DRE urine samples was approved by the US FDA in 2012 (Rittenhouse et al., 2013[26]). Nevertheless, this assay based on target captured, transcription-mediated amplification and hybridization is not applicable for routine screening because of its cost and complexity.

In this study, the MNP-based PCR in combination with colorimetric detection was successfully developed for *PCA3 *detection. MNPs have advantageous properties due to their small size, high surface area, high stability, and ease of surface functionalization (Zhu et al., 2018[40]). Functionalized MNPs coated with antibodies or oligonucleotides have been widely used for the purification, extraction and detection of biomolecules (Chan et al., 2008[8]; Sandhu et al., 2010[28]). Herein, the MNPs were immobilized on *PCA3* forward primer. The binding efficiency of the immobilization was approximately 51.9 %. The efficiency of immobilization is in good agreement with the result of a previous study (Tangchaikeeree et al., 2017[35]). In the literature, excess MNPs inhibit the PCR reaction by adsorbing to PCR components and DNA templates and the inhibition was concentration-dependent (Bai et al., 2015[3]). It has been demonstrated that the amplification of the PCR products was completely inhibited when the amount of MNPs exceeded 20 μg. Therefore, the optimal concentration of MNP conjugated-forward primer was examined. As a result, positive control using 6 μg of MNP conjugated-forward primer showed the highest relative OD_450_ that was approximately 3-fold greater than that of negative control. There was no influence of MNPs on PCR reaction at this concentration. 

Significantly, the developed method was extremely specific for the *PCA3 *target gene. It has been previously evident that the expression of *PCA3 *was found in cell lines that are androgen-dependent, such as LNCaP cells (Lemos et al., 2016[17]). Our results showed that the relative OD_450_ of LNCaP was statistically higher than that of other cell lines (*P*-value < 0.05). Of note, there is no false positive when conducting the test using cDNA from normal prostate cell line (RWPE-1) as well as other cancer cell lines (MDA-MB-436 and K562 cell lines). As expected, the results from the MNP-based colorimetric assay are consistent with the results from gel electrophoresis. These finding indicated that the primers for *PCA3* amplification in this study are very specific. In addition, the sensitivity of detection was investigated. As a result, the significant differences were observed between the relative OD_450_ of positive samples (1 µg to 1 fg of cDNA template) and negative control. The detection limit of the assay was approximately 1 fg of the template which was 1,000-fold lower than the conventional RT-PCR followed by gel electrophoresis. These results suggested that our method is very sensitive. The MNPs serve as a solid support to separate unbound reagents, such as free primers and reagents that are used in detection system. Every MNP can convey thousands of copies of PCR products which in turn increase sensitivity. Recently, a similar technique has been applied for detection of gene expression in chronic myeloid leukemia, bacteria and parasite with the detection limits ranging from picogram to femtogram levels of template (Manthawornsiri et al., 2016[20]; Jansaento et al., 2016[15]; Tangchaikeeree et al., 2017[35]). Our results are in good agreement with these previous studies which indicated that this assay provides the accuracy and reliability for detection of the gene expression. The assay time after PCR of our method (only 1 h) was much shorter than that of previous MNP-based colorimetric assays (>1 h) (Manthawornsiri et al., 2016[20]; Jansaento et al., 2016[15]; Tangchaikeeree et al., 2017[35]). This could be due to our assay used the 3,3′,5,5′-tetramethylbenzidine (TMB) as a substrate instead of 2,2'-azino-bis(3-ethylbenzothiazoline-6-sulphonic acid) (ABTS). So far as we know, ABTS is less sensitive than TMB and it may take some time to properly develop its color. Moreover, our results showed 2-times higher relative absorbance of the colorimetric signaling obtained from the MNP-based PCR products compared to previous studies (Manthawornsiri et al., 2016[20]; Tangchaikeeree et al., 2017[35]). This significantly higher sensitivity could be granted from the advantages of high sensitive TMB substrate and using of dual biotin molecules, the latter of which showed that they can increase binding strength with streptavidin as reported elsewhere (Yuce et al., 2014[39]). 

To examine the potential practicality of the method, *PCA3 *detection in fifteen urine samples using the developed assay and the qRT-PCR were carried out. The results of our assay showed a good agreement with the qRT-PCR results. The method could detect the significant differences of the relative OD_450 _of *PCA3* products among PCa patients, BPH patients and healthy controls. The results indicated that our assay can discriminate PCa patients from other groups. Notably, the relative *PCA3* expression of PCa patients detected by our assay was not well correlated with the relative *PCA3* expression level determined by qRT-PCR. Nevertheless, our assay is feasible and applicable to use as a screening method for PCa diagnosis with high specificity and sensitivity. In the literature, the nanomaterial-based assays for *PCA3* detection were recently developed. For example, the colorimetric method for *PCA3* detection in urine using unmodified gold nanoparticles (AuNPs) and the thio-labeled PCR primer was established by our group (Htoo et al., 2019[13]). In the present study, it revealed that the MNP-based colorimetric method exhibited the better sensitivity than the previous one. Another method using AuNPs combined with the surface-enhanced Raman scattering (SERS) technique and the lateral flow assay (LFA) platform was created to detect the *PCA3 *mimic DNA in serum (Fu et al., 2019[12]). The limit of detection of the assay was estimated to be 3 fM. Obviously, the detection limit of our method is comparable. However, our method is simpler, cost-effective and less tedious in terms of platform preparation. In addition, it is not required for sophisticated instrument to detect the signal.

## Conclusion

In summary, the MNP-based PCR coupled with colorimetric enzyme-linked oligonucleotide assay has been developed. The proposed method is based on combining the MNPs-labeled forward primer and the dual biotinylated reverse primer in target gene amplification. Subsequently, an enzyme-substrate system is employed for detection step. This assay is rapid, simple and cost-effective. The assay time after PCR can be accomplished within an hour. Our assay provided high specificity for *PCA3 *when testing with* PCA3 *positive and negative cell lines. The high sensitivity was also obtained with the detection limit at the femtogram level of template. Importantly, this assay is applicable with potential use in detection of urinary *PCA3* for PCa. This technique can distinguish PCa patients from BPH patients and healthy controls based on various expression levels of *PCA3 *in urine sediments of subjects. It should be noted that this method demonstrated good performance compared with qRT-PCR for *PCA3* detection in urine samples. Taken together, the developed assay is promising and feasible for clinical practice. Finally, our assay could be useful as a diagnostic method for screening of prostate cancer.

## Conflict of interest

The authors declare that they have no financial or commercial conflict of interest.

## Acknowledgements

This work was supported by National Research Council of Thailand and Health Systems Research Institute (grant number HSRI 60-026). Khin Phyu Pyar Htoo was supported by the Mahidol-Norway Capacity Building Initiative for ASEAN Scholarship.

## Figures and Tables

**Table 1:**
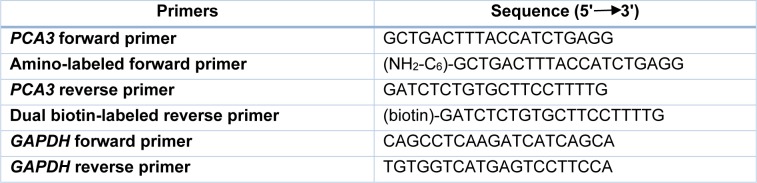
List of primers

**Table 2:**
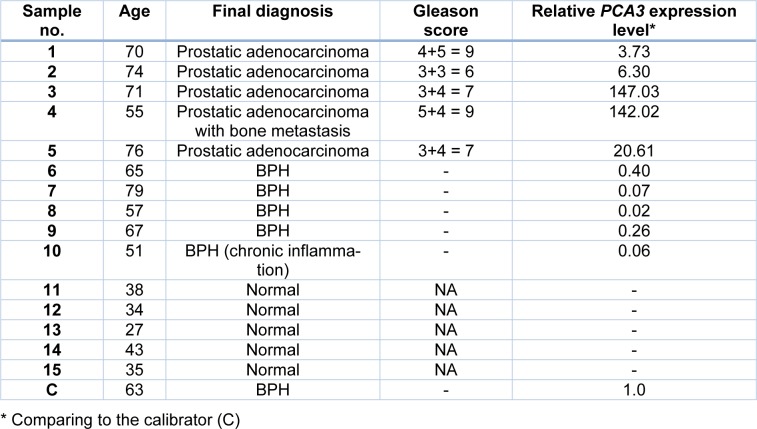
Characteristics of participants
